# Long-term wholegrain intake in line with the Nordic Nutrition Recommendations 2023 and risk of breast cancer in a population-based cohort of women

**DOI:** 10.1007/s00394-026-04013-8

**Published:** 2026-06-19

**Authors:** Stephanie Pitt, Niclas Håkansson, Joanna Kaluza, Susanna C. Larsson, Agneta Åkesson, Alicja Wolk

**Affiliations:** 1 Institute of Environmental Medicine (IMM), Karolinska Institutet, 17 177 Stockholm, Sweden; 2 Department of Human Nutrition, Warsaw, University of Life Sciences–SGGW, 02-787 Warsaw, Poland; 3 Medical Epidemiology, Department of Surgical Sciences, Uppsala University, Uppsala, Sweden

**Keywords:** Wholegrain cereals, Breast cancer, Nordic Nutrition Recommendations, Swedish cohort

## Abstract

**Purpose:**

Current findings on the relationship between wholegrain intake and breast cancer are inconsistent. We aimed to estimate the association between long-term wholegrain intake and breast cancer risk, specifically investigating: (i) adherence to the updated Nordic Nutrition Recommendations (NNR2023) guidelines on wholegrain intake, and (ii) consumption of specific wholegrain products.

**Methods:**

Data from food frequency questionnaires were used to assess adherence to NNR2023 guidelines on wholegrain intake and consumption of wholegrain products among 36,479 women (48–83 years) in the Swedish Mammography Cohort at two timepoints. Time-updated Cox proportional hazards regression models were used to estimate multivariable-adjusted hazard ratios (HR) and 95% confidence intervals (CI) for incident total breast cancer and hormone receptor-positive and -negative subtypes.

**Results:**

During a mean follow-up of 16.5 years, 1,979 breast cancer cases were identified. Compared to long-term low adherence (< 50%, corresponding to < 45 g/day wholegrain intake), the HRs (95% CIs) for women with full adherence (≥ 90 g/day intake) were 0.78 (0.66, 0.94), 0.82 (0.67, 1.03), and 1.08 (0.65, 1.80), for total breast cancer, hormone receptor-positive, and hormone receptor-negative breast cancer, respectively. There was no clear association between long-term consumption of any specific wholegrain products and total breast cancer risk, although HRs for high oatmeal and for high breakfast cereal consumption were < 1, whilst HRs for high crispbread consumption were > 1.

**Conclusion:**

Wholegrain intake in line with NNR2023 was associated with a lower risk of total breast cancer. However, different wholegrain products may be differently associated with breast cancer risk, indicating the need for further investigation.

**Supplementary Information:**

The online version contains supplementary material available at 10.1007/s00394-026-04013-8.

## Introduction

The development of breast cancer—the most prevalent cancer globally [1]—is closely related to levels of endogenous steroid hormones, chiefly oestrogen and progesterone, as well as exogeneous sources. High fibre diets have been postulated to lower breast cancer risk by reducing reabsorption of oestrogen in the colon [2] thus reducing its circulating levels [3]. Accordingly, an inverse association between high fibre intake and risk of breast cancer has often been reported [3–5]. Given that wholegrain foods are generally high in fibre, it is plausible that high intake of wholegrain cereals may indeed contribute to reduced breast cancer risk. However, current findings on an association between wholegrain cereal intake and breast cancer risk are inconsistent [6–10]. Meta-analyses [11] are also limited by large variation in the types of grains consumed among different populations, leading to high heterogeneity between studies. Inconsistent findings on wholegrain and breast cancer may also be partially due to a lack of consideration on the source(s) of wholegrain within a single wholegrain term. Different grains have different compositions including different bioactive compounds and other potential anti-cancer properties [12–14], as well as varying amounts and types of dietary fibre [12, 15], which may lead to distinct influences on circulating levels of steroid hormones.

Nonetheless, the updated Nordic Nutrition Recommendations 2023 (NNR2023) guidelines advise a wholegrain intake of at least 90 g/day to confer health benefits [16]. As such, the aim of the present study was to estimate the association between long-term wholegrain intake at different levels of adherence to the NNR2023 guidelines, with risk of total breast cancer and hormone receptor-positive and -negative subtypes. Furthermore, given the abovementioned difference between grain species, we further aimed to estimate the association between consumption of specific wholegrain products composed of different grains (e.g., oat, wheat, and rye) and breast cancer risk.

## Methods

### Study population

The Swedish Mammography Cohort (SMC) [17] was established in 1987–1990 when 90,303 women born between 1914–48 and living in central Sweden (Uppsala or Västmanland counties) were invited to participate in a mammography screening program and to complete a food frequency questionnaire (FFQ) (74% response rate). In the late autumn of 1997, 56,030 participants still alive and residing in the area were sent an extended questionnaire on sociodemographic and lifestyle factors, including a 96-item FFQ. The 1997 FFQ was completed by 39,227 women. The information collected in 1997 was used as baseline in the present study, due to more comprehensive information on covariates and because breast cancer subtype data was not available prior to 1997. Re-investigation occurred in 2008/09, whereby a further health questionnaire and 149-item FFQ was sent to all participants who had responded to the 1997 FFQ and were still alive at the time of re-investigation. The 2009 FFQ was completed by 25,259 women. From the 39,227 women who completed the FFQ in 1997, the following exclusions were made: incorrect or missing identification (*n* = 243); death prior to baseline (*n* = 42); pre-baseline cancer diagnosis (*n* = 1,811); implausible energy intake (*n* = 481); missing response to all four wholegrain products (*n* = 171).

As such, a total of 36,479 women were included in the analytical cohort. Ethical approval was obtained for SMC by the Swedish Ethical Review Authority, and consent was given by completing and returning the questionnaire.

### Exposure assessment

Wholegrain intake and consumption of wholegrain products were assessed using FFQ responses in both 1997 and 2009. In both FFQs, participants reported usual consumption of each food/drink as either a predefined frequency—*per month* (0 or 1–3), *per week* (1–2, 3–4, or 5–6), or *per day* (1, 2, or 3 +)—or for some foods (e.g., slices of bread) as an open-ended question. Missing data (i.e., consumption not reported) was assumed to be non-consumption. Frequencies were converted into daily servings and in g/day using age-specific portion sizes determined from weighted food records of an SMC subgroup. The 1997 FFQ has been previously validated for consumption of wholegrain products, with Spearman correlation coefficients ranging from 0.5 to 0.7 [18]. Furthermore, the study population arises from central Sweden where wholegrain is the dominant source of fibre [19].

Total wholegrain intake was estimated by summing up reported consumption of wholegrain products (g/day) multiplied by the respective percentage of wholegrain in each product (i.e., wholegrain as an ingredient). In line with Ross et al. [20], we determined the percentage of wholegrain in each product when “ready-to-eat”, with percentages obtained from the Swedish Food Agency [21]. We chose to investigate three levels of adherence to NNR2023 guidelines on wholegrain intake: i) low adherence (< 50%, corresponding to < 45 g/day); ii) partial adherence (≥ 50 to < 100%, corresponding to ≥ 45 to < 90 g/day); and iii) full adherence (corresponding to ≥ 90 g/day).

Wholegrain products were defined as FFQ items containing at least 50% wholegrain by dry weight (i.e., excluding water) [22], based on previous literature and as reported in NNR2023. An exception was made for breakfast cereals, which was a composite of both muesli and other breakfast cereals in the 1997 FFQ, but was subsequently split into two questions in 2009. As such, in 1997, the item includes only 45% wholegrain by dry weight, but > 50% wholegrain in 2009 (Supplementary Table 1). In total, there were four wholegrain products in the FFQs: oatmeal, breakfast cereals, soft wholegrain bread, and crispbread. Furthermore, three additional wholegrain products (wholegrain rice, wholegrain pasta, and bulgar/cous cous) were included in the 2009 FFQ. Whilst these contributed to total wholegrain intake in 2009, consumption of the specific additional wholegrain products were not investigated individually due to lack of information in 1997. Long-term wholegrain intake and consumption of wholegrain products were estimated using the cumulative average between the 1997 and 2009 FFQ responses. In the case of non-response to the 2009 FFQ, values from 1997 were carried forward.

### Cases ascertainment

Information about histologically confirmed incident breast cancer cases was obtained through linkage of SMC to the Swedish Cancer Register, with essentially complete case ascertainment [23]. Data on breast cancer subtype by receptor status were obtained by linking SMC to the Swedish National Quality Registry for Breast Cancer [24]. We considered total breast cancer cases and cases of oestrogen and progesterone receptor-positive (ER+/PR+) and oestrogen and progesterone receptor-negative (ER-/PR-) cancer. Date of death was obtained through linkage of SMC to the Swedish Registry of Causes of Death.

### Covariate assessment

Information on age and education was obtained from the 1997 questionnaire. Further information on smoking status, walking/cycling, exercise, weight, height, alcohol consumption, consumption of refined grain products, and energy intake were obtained from both the 1997 and 2009 questionnaire. Information on family history of breast cancer, age at menarche, parity, ever use of hormone replacement therapy, ever use of contraceptive pills, and menopause status was also obtained from the 1997 questionnaire. Body Mass Index (BMI) was calculated from reported information on weight and height in kg/m^2^. Dietary quality was assessed using an adapted version of the modified Mediterranean Diet Score (mMDS) [25] and based on FFQ responses. After excluding the wholegrains and alcohol components (see below), the mMDS was determined with 1 point scored for consumption above the median of fruit/vegetables, legumes/nuts, fermented dairy, fish, and any use of olive or rapeseed oil; and 1 point for consumption below the median of red/processed meat. Participants were subsequently categorised to low (0–2 points), medium (3–4 points), or high (5–6 points) adherence to a Mediterranean-style diet. The maximum score was 6 instead of 8 as both the wholegrain and alcohol [26] components were not included to avoid over-adjustment in the statistical models. Total energy intake (kcal/day) was calculated by summing up the energy (kcal) per gram of each FFQ item (obtained from food tables of the Swedish Food Agency [21]) multiplied by daily consumption in grams. Missing covariate data in 1997 were included as a missing indicator category. Missing indicator categories were retained for information missing again at re-investigation, otherwise missing information at re-investigation in 2009 was replaced by information from 1997 (i.e., last observation carried forward).

### Statistical analysis

Participants were followed from baseline on 01-January-1998 until breast cancer diagnosis, death, or the end of follow up on 31-Decmber-2016, whichever came first. Time-updated Cox proportional hazards regression models with time in the study as the underlying time scale were used to assess the association of (i) total wholegrain intake (g/day) at three levels of adherence to NNR2023; and (ii) consumption of four wholegrain products: oatmeal, breakfast cereals, soft wholegrain bread, and crispbread (servings/week, categorical), with subsequent breast cancer diagnosis. For the exposure (wholegrain intake and consumption of wholegrain products) time-updated cumulative average was used, whilst all other covariates in the model (excluding education) were time-updated at the point of re-investigation in 2009 for those participants who remained in the analysis beyond 01-July-2009. Separate models were used for total, ER+/PR+ and ER-/PR- subtypes.

Three analytical models were employed. All variables included in the models were pre-specified based on having known or likely associations with both wholegrain intake and breast cancer or are strong predictors of breast cancer and were assessed using a directed acyclic graph. Model 1 was minimally adjusted, including age only. In Model 2, adjustments were made for the following confounders: age, education level (≤ 9 years, 10–11 years, or ≥ 12 years), smoking status (never, former, and current), walking/cycling (< 20 min/day, ≥ 20 and < 60 min/day, or ≥ 60 min/day), exercise (≤ 1 h/week, 2–3 h/week, or ≥ 4 h/week), body mass index (BMI) (< 25 kg/m^2^, ≥ 25 and < 27.5 kg/m^2^, ≥ 27.5 and < 30 kg/m^2^, or ≥ 30 kg/m^2^), family history of breast cancer (yes/no), alcohol consumption (continuous, g/day), consumption of refined grain foods (continuous, g/day), modified Mediterranean Diet Score (mMDS) (low (0–2 points), medium (3–4 points), or high (5–6 points)), and energy intake (continuous, kcal/day). In Model 3, in addition to the adjustments in Model 2, we further adjusted for the following breast cancer risk factors: age at menarche (≤ 12 years, ≥ 13 years), parity (nulliparous, 1–2 children, ≥ 3 children), age at first birth (nulliparous, < 31 years, ≥ 31 years), ever use of hormone replacement therapy (yes/no), ever use of contraceptive pills (yes/no), menopause status and age of occurrence (pre-menopausal, post-menopausal and occurred at < 51 years, post-menopausal and occurred at ≥ 51 years). Model 2 was selected as the main model. Tests for trends were performed using the median value in category (for total wholegrain intake and consumption of wholegrain products, respectively) and including this in the model as a continuous variable. All analyses were performed using Stata 16.1, with two-sided significance set to *p* = 0.05. The assumption of proportional hazards was met, as indicated by the Schoenfeld residuals (*p* > 0.05). Finally, three sensitivity analyses were conducted. First, all analyses were repeated using only baseline information from 1997 (i.e., non-time-updated). In a second sensitivity analysis, we included only participants with complete data on the exposure at baseline (i.e., those that responded to all four questions on wholegrain products in 1997). Last, analyses were repeated upon excluding the three additional wholegrain products assessed only in 2009 (wholegrain rice, wholegrain pasta, and bulgar/cous cous) from the wholegrain intake term. All results are presented as hazard ratios (HR) with 95% confidence intervals (CI).

## Results

**Fig. 1:**
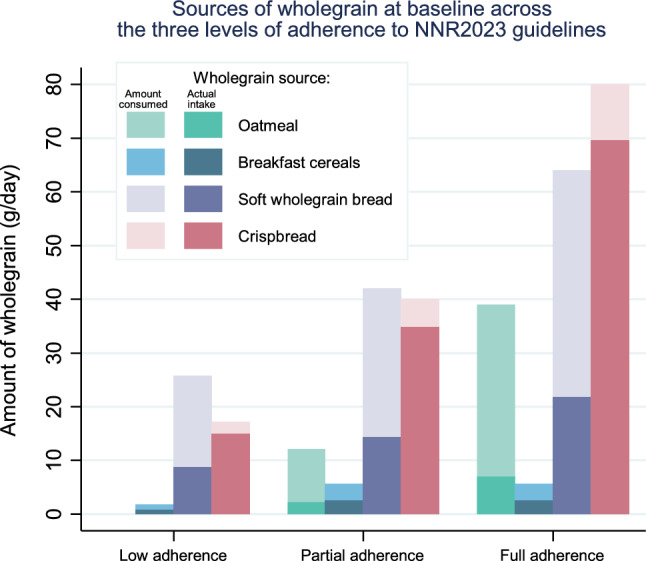
Median consumption of wholegrain products “ready-to-eat” (light shaded bars) and corresponding wholegrain intake (i.e., the proportion of the product that is wholegrain; dark shaded bars) (both g/day) at baseline, according to each level of adherence to the Nordic Nutrition Recommendations (NNR) 2023 guidelines on wholegrain intake: low adherence (< 50%, corresponding to < 45 g/day); partial adherence (50 to < 100%, corresponding to ≥ 45 to < 90 g/day); and full adherence (100%, corresponding to ≥ 90 g/day)

A total of 1,979 breast cancer cases were diagnosed over a mean of 16.5 years of follow-up and 602,248 person-years. We had information on ER and PR status for 1,842 women, with 1,239 cases (67%) ER+/PR+ and 204 (11%) ER-/PR-. The mean (± SD) age of all participants was 62.3 years (9.2) and the vast majority (81%) of them were postmenopausal at baseline. Breast cancer cases were slightly younger at baseline (mean 60.8 years (8.5)). In general, compared to low adherence, women with high adherence to NNR2023 wholegrain recommendations (100% vs.  < 50%, corresponding to ≥ 90 versus  < 45 g/day, wholegrain intake, respectively), had slightly more beneficial lifestyle habits, whilst other age-standardised baseline characteristics did not vary substantially across the three levels of adherence (Table 1). The majority of total wholegrain intake at baseline came from consumption of crispbread at all three levels of NNR2023 adherence (Fig. 1).

**Table 1:** Age-standardised characteristics of 36,479 women in the Swedish Mammography Cohort (SMC) at baseline in 1997

Characteristic	Wholegrain intake^a^ in relation to NNR2023		
	Low adherence	Partial adherence	Full adherence
Percentage of adherence to NNR2023	< 50%	50 to < 100%	≥ 100%
Range of wholegrain intake (g/day)	0 to < 45	≥ 45 and < 90	≥ 90
Participants, *n*	15,369	16,980	4,130
Breast cancer cases, *n*	860	939	180
Age in years, mean (SD)	61.2 (9.0)	63.0 (9.3)	63.4 (9.4)
*Years of education, %*			
≤ 9	71.2	75.0	77.0
10–11	7.9	7.4	6.7
≥ 12	20.6	17.4	15.0
Missing	0.3	0.3	0.4
*Smoking status, %*			
Never	49.0	55.8	56.2
Former	24.0	21.6	21.4
Current	25.3	21.0	20.4
Missing	1.8	1.7	2.0
*Walking/cycling, minutes/day, %*			
< 20	30.8	25.5	24.2
≥ 20 and < 60	46.9	51.0	46.5
≥ 60	13.7	16.7	20.2
Missing	8.6	6.8	9.0
*Exercise, hours/week, %*			
≤ 1	43.5	36.2	31.2
2–3	28.1	31.9	30.7
≥ 4	17.0	22.2	26.7
Missing	11.4	9.6	11.4
BMI, mean (SD)	25.1 (4.0)	25.0 (3.9)	24.8 (4.0)
Family history of breast cancer, %	9.3	9.2	8.9
Alcohol, g/day, mean (SD)	1.4 (0.6)	1.3 (0.5)	1.3 (0.5))
Refined grain products, g/day, median (p5, p95)	64 (18, 152)	59 (10, 141)	59 (10, 163)
mMDS points, mean (SD)	2.8 (1.4)	3.2 (1.4)	3.3 (1.4)
Energy intake^b^, kcal/day, mean (SD)	1500 (450)	1800 (500)	2200 (550)
*Age at menarche, %*			
≤ 12 years	26.8	24.4	23.2
≥ 13 years	60.7	63.6	64.5
Missing	12.5	12.0	12.3
*Parity, %*			
Nulliparous	7.3	7.3	7.3
1–2 children	57.5	56.1	54.6
≥ 3 children	32.7	34.0	35.4
Missing	2.5	2.7	2.7
*Age at first birth, %*			
Nulliparous	7.3	7.3	7.3
< 31 years	81.7	81.3	81.2
≥ 31 years	7.2	7.6	7.2
Missing	3.9	3.8	4.3
Ever use of hormone replacement therapy, %	45.9	46.0	43.8
Ever use of contraceptive pills, %	47.5	40.6	36.5
*Menopause status, %*			
Pre-menopausal	21.0	17.2	18.0
Post-menopausal, occurring at < 51 years	43.3	43.2	43.5
Post-menopausal, occurring at ≥ 51 years	34.8	38.7	37.8
Missing	1.0	0.9	0.7
*Consumption of wholegrain products, servings/week, median (p5, p95)*			
Oatmeal	0 (0, 3.5)	0.5 (0, 7)	1.5 (0, 7)
Breakfast cereals	0.5 (0, 7)	1.5 (0, 7)	1.5 (0, 7)
Soft wholegrain bread	5 (0, 14)	7 (0, 21)	14 (0, 49)
Crispbread	6 (0, 14)	14 (3, 22)	28 (8, 56)

Missing information on BMI (3.4%), ever use of hormone replacement therapy (3.4%), and ever use of contraceptive pills (3.2%). Missing in consumption of oatmeal (13.3%), breakfast cereals (19.5%), soft wholegrain bread (15.2%), and crispbread (8.2%) assumed as non-consumption

*NNR* Nordic Nutrition Recommendations, *BMI* Body Mass Index, *SD* Standard Deviation, *mMDS* modified Mediterranean Diet Score, p5: 5th percentile; p95: 95th percentile

^a^Wholegrain intake was determined as the sum of the wholegrain ingredients included in several wholegrain food products

^b^Rounded to nearest 50 kcal

### Wholegrain intake in line with NNR2023 and breast cancer risk

Under multivariable-adjusted Model 2 in time-updated analyses, compared to women with low long-term adherence to NNR2023 guidelines on wholegrain intake, the HR of total breast cancer was 0.78 (95% CI 0.66, 0.94) among women with full adherence (Table 2). Compared to low adherence, there was no association between wholegrain intake with partial adherence (> 50% but < 100%) and total breast cancer (HR: 1.01; 95% CI 0.91, 1.11). Regarding ER+/PR+ breast cancer, the point estimate for the HR for women with full adherence was of the same direction and similar magnitude as for total breast cancer, although the association was somewhat attenuated (HR: 0.82; 95% CI 0.67, 1.03), whilst no association between full adherence and ER-/PR- breast cancer was observed (HR: 1.08; 95% CI 0.65, 1.80). No substantial difference in point estimate or confidence intervals were observed between results from Model 2 and Model 3 for total breast cancer, or breast cancer subtypes. In non-time-updated sensitivity analyses—in which no information on the exposure or covariates was updated—the observed association between full adherence at baseline and total breast cancer remained and was of a similar magnitude, with point estimates and 95% CIs for the studied breast cancer subtypes also of the same direction and similar magnitude to the main analysis (Supplementary Table 2). A slightly stronger association between full adherence and total breast cancer was observed in time-updated complete data analysis (including 22,084 women and 1,220 total breast cancer cases), in which participants were additionally excluded from the analysis upon complete non-response to the FFQ at re-investigation (HR: 0.74; 95% CI 0.60, 0.93) (Model 2–Supplementary Table 3). Finally, the findings were unchanged upon excluding the three additional wholegrain products assessed only in 2009 within the wholegrain intake term (results not shown).

**Table 2:** Hazard Ratio (HR) and 95% Confidence Intervals (CI) between time-updated wholegrain intake^a^ in relation to NNR2023 guidelines and risk of breast cancer by receptor-defined subtype, in a population-based cohort of Swedish women (*n* = 36,479), totalling 602,248 person-years

Wholegrain intake^a^ in relation to NNR2023				Model 1 HR (95% CI)	Model 2 HR (95% CI)	Model 3 HR (95% CI)
Level of adherence	Percentage of adherence	Intake range (g/day)	Number of cases			
			*N* = 1,979	*Total breast cancer*		
Low	< 50%	0 to < 45	812	Ref	Ref	Ref
Partial	≥ 50% and < 100%	≥ 45 to < 90	988	1.02 (0.92, 1.11)	1.01 (0.91, 1.11)	1.01 (0.91, 1.11)
Full	100%	≥ 90	179	0.80 (0.68, 0.94)	0.78 (0.66, 0.94)	0.79 (0.66, 0.94)
		*p*-trend		0.03	0.03	0.04
			*N* = 1,239	*ER + /PR + *		
Low	< 50%	0 to < 45	492	Ref	Ref	Ref
Partial	≥ 50% and < 100%	≥ 45 to < 90	635	1.07 (0.95, 1.21)	1.07 (0.94, 1.21)	1.07 (0.94, 1.21)
Full	100%	≥ 90	112	0.83 (0.68, 1.02)	0.82 (0.67, 1.03)	0.83 (0.66, 1.04)
		*p*-trend		0.27	0.27	0.31
			*N* = 204	*ER-/PR-*		
Low	< 50%	0 to < 45	77	Ref	Ref	Ref
Partial	≥ 50% and < 100%	≥ 45 to < 90	102	1.10 (0.81, 1.48)	1.06 (0.78, 1.46)	1.08 (0.79, 1.48)
Full	100%	≥ 90	25	1.17 (0.74, 1.84)	1.08 (0.65, 1.80)	1.08 (0.65, 1.80)
		*p*-trend		0.45	0.72	0.72

Analysis was time updated at the point of re-investigation in 2009, meaning that all participants that remained in the analysis received an updated value of the exposure and covariates from that timepoint (except for education level, family history of breast cancer, and further adjustments in Model 3). Any missing information at re-investigation was carried forward from the previous investigation. Missing data at baseline were included as a missing indicator category for: education (0.3%), smoking status (1.8%), walking/cycling (7.8%), exercise (10.6%), BMI (3.4%), age at menarche (12.3%), parity (11.1%), ever use of hormone replacement therapy (3.4%), ever use of contraceptive pills (3.2%), menopause status (0.9%). Missing data in 2009 were included as a missing indicator category for: smoking status (0.9%), walking/cycling (3.3%), exercise (4.6%), and BMI (1.6%)

*NNR* Nordic Nutrition Recommendations, *ER+/PR+* oestrogen receptor-positive/progesterone receptor-positive, *ER-/PR-* oestrogen receptor-negative/progesterone receptor-negative

Model 1: age-adjusted only

Model 2: multivariable adjusted model, with adjustments for: age, education level (≤ 9 years, 10–11 years, or ≥ 12 years), smoking status (never, former, and current), walking/cycling (< 20 min/day, ≥ 20 and < 60 min/day, or ≥ 60 min/day), exercise (≤ 1 h/week, 2–3 h/week, or ≥ 4 h/week), body mass index (BMI) (< 25 kg/m^2^, ≥ 25 and < 27.5 kg/m^2^, ≥ 27.5 and < 30 kg/m^2^, or ≥ 30 kg/m^2^), family history of breast cancer (yes/no), alcohol consumption (continuous, g/day), consumption of refined grain foods (continuous, g/day), modified Mediterranean Diet Score (mMDS) (low (0–2 points), medium (3–4 points), or high (5–6 points)), and energy intake (continuous, kcal/day)

Model 3: same as Model 2 with additional adjustments for: age at menarche (≤ 12 years, ≥ 13 years), parity (nulliparous, 1–2 children, ≥ 3 children), age at first birth (nulliparous, < 31 years, ≥ 31 years), ever use of hormone replacement therapy (yes/no), ever use of contraceptive pills (yes/no), menopause status and age of occurrence (pre-menopausal, post-menopausal and occurred at < 51 years, post-menopausal and occurred at ≥ 51 years)

Person-time (years) per level of adherence: low: 224,283; partial: 290,660; full: 67,305

^a^Wholegrain intake was determined as the sum of the wholegrain ingredients from several wholegrain food products

### Consumption of wholegrain products and breast cancer risk

**Fig. 2:**
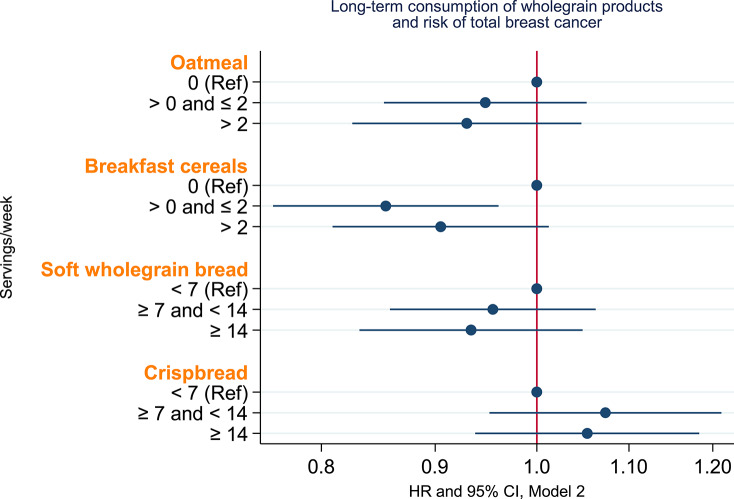
Hazard Ratios (HRs) and 95% Confidence Interval (CI) of total breast cancer for three groups of consumption of wholegrain products among 36,479 women in the Swedish Mammography Cohort

The direction of point estimates for the HRs of breast cancer and its subtypes varied between the wholegrain products studied (Fig. 2). In time-updated multivariable-adjusted models, no clear association with total breast cancer or either studied subtype was observed for long-term oatmeal consumption (> 2 servings/week *vs* no consumption), although all point estimates were < 1 (Table 3). Compared to non-consumers, moderate consumption (> 0 to ≤ 2 servings/week) of breakfast cereals was associated with a lower risk of total breast cancer (in Model 2–HR: 0.86; 95% CI 0.76, 0.96), with an indication of lower risk observed among high consumers (> 2 servings/week, HR: 0.91; 95% CI 0.81, 1.01). Soft wholegrain bread was not clearly associated with total breast cancer or either studied subtype (≥ 14 vs.  < 7 servings/week) in either multivariable-adjusted Model 2 or Model 3. Of note, consumption of crispbread (≥ 14 vs.  < 7 servings/week) was associated with a higher risk of ER-/PR- breast cancer (in Model 2–HR: 1.56; 95% CI 1.06, 2.28). In both non-time-updated sensitivity analyses (Supplementary Table 4) and time-updated complete data analysis (Supplementary Table 5), all point estimates were of the same direction and similar magnitude to that of the main analysis.

**Table 3:** Hazard Ratio (HR) and 95% Confidence Intervals (CI) between time-updated consumption of wholegrain products and risk of breast cancer by receptor-defined subtype, in a population-based cohort of Swedish women (*n* = 36,479), totalling 602,248 person-years

Consumption of wholegrain products^a^ (servings/week)		Total breast cancer (cases *n* = 1,979)			ER + /PR + (cases *n* = 1,239)			ER-/PR- (cases *n* = 204)		
	Person-years	Model 1 HR (95% CI)	Model 2 HR (95% CI)	Model 3 HR (95% CI)	Model 1 HR (95% CI)	Model 2 HR (95% CI)	Model 3 HR (95% CI)	Model 1 HR (95% CI)	Model 2 HR (95% CI)	Model 3 HR (95% CI)
*Oatmeal*										
0	242,832	Ref	Ref	Ref	Ref	Ref	Ref	Ref	Ref	Ref
> 0 and ≤ 2	202,930	0.94 (0.85, 1.05)	0.95 (0.85, 1.05)	0.95 (0.86, 1.06)	0.95 (0.84, 1.09)	0.94 (0.83, 1.08)	0.95 (0.83, 1.08)	0.92 (0.67, 1.28)	0.91 (0.66, 1.26)	0.92 (0.66, 1.28)
> 2	156,486	0.91 (0.81, 1.02)	0.93 (0.83, 1.05)	0.93 (0.83, 1.05)	0.94 (0.82, 1.09)	0.95 (0.81, 1.10)	0.94 (0.81, 1.09)	0.84 (0.59, 1.21)	0.82 (0.57, 1.19)	0.82 (0.57, 1.19)
*p-*trend		0.20	0.37	0.36	0.56	0.64	0.61	0.42	0.37	0.35
*Breakfast cereals*										
0	188,889	Ref	Ref		Ref	Ref	Ref	Ref	Ref	Ref
> 0 and ≤ 2	177,712	0.88 (0.78, 0.99)	0.86 (0.76, 0.96)	0.85 (0.75, 0.95)	0.84 (0.73, 0.97)	0.82 (0.71, 0.95)	0.82 (0.70, 0.94)	0.76 (0.53, 1.09)	0.74 (0.51, 1.06)	0.75 (0.52, 1.07)
> 2	235,640	0.93 (0.84, 1.04)	0.91 (0.81, 1.01)	0.88 (0.79, 0.99)	0.90 (0.78, 1.03)	0.89 (0.77, 1.02)	0.86 (0.75, 1.00)	0.77 (0.55, 1.07)	0.74 (0.52, 1.04)	0.74 (0.52, 1.05)
*p-*trend		0.68	0.49	0.27	0.49	0.54	0.33	0.32	0.26	0.25
*Soft wholegrain bread*										
< 7	227,569	Ref	Ref		Ref	Ref	Ref	Ref	Ref	Ref
≥ 7 and < 14	194,132	0.97 (0.87, 1.07)	0.95 (0.87, 1.06)	0.95 (0.85, 1.06)	0.94 (0.82, 1.07)	0.93 (0.81, 1.06)	0.93 (0.81, 1.06)	1.29 (0.93, 1.80)	1.29 (0.92, 1.80)	1.29 (0.92, 1.80)
≥ 14	180,547	0.95 (0.85, 1.06)	0.94 (0.83, 1.05)	0.93 (0.83, 1.04)	0.91 (0.79, 1.05)	0.90 (0.78, 1.05)	0.90 (0.77, 1.04)	1.18 (0.83, 1.67)	1.17 (0.81, 1.68)	1.17 (0.81, 1.68)
*p-*trend		0.29	0.26	0.33	0.18	0.17	0.15	0.40	0.45	0.44
*Crispbread*										
< 7	160,106	Ref	Ref		Ref	Ref	Ref	Ref	Ref	Ref
≥ 7 and < 14	171,947	1.07 (0.95, 1.21)	1.07 (0.95, 1.21)	1.08 (0.95, 1.21)	1.05 (0.90, 1.22)	1.04 (0.90, 1.22)	1.05 (0.90, 1.22)	1.29 (0.86, 1.93)	1.30 (0.86, 1.94)	1.31 (0.87, 1.96)
≥ 14	270,195	1.05 (0.94, 1.17)	1.06 (0.94, 1.19)	1.07 (0.95, 1.20)	1.07 (0.93, 1.23)	1.07 (0.93, 1.24)	1.08 (0.93, 1.25)	1.55 (1.08, 1.24)	1.56 (1.06, 2.28)	1.58 (1.08, 2.30)
*p-*trend		0.57	0.49	0.28	0.46	0.37	0.30	0.019	0.023	0.022

Analysis was time updated at the point of re-investigation in 2009, meaning that all participants that remained in the analysis received an updated value of the exposure and covariates from that timepoint (except for education level, family history of breast cancer, and further adjustments in Model 3). Any missing information at re-investigation was carried forward from the previous investigation. Missing data at baseline were included as a missing indicator category for: education (0.3%), smoking status (1.8%), walking/cycling (7.8%), exercise (10.6%), BMI (3.4%), age at menarche (12.3%), parity (11.1%), ever use of hormone replacement therapy (3.4%), ever use of contraceptive pills (3.2%), menopause status (0.9%). Missing data in 2009 were included as a missing indicator category for: smoking status (0.9%), walking/cycling (3.3%), exercise (4.6%), and BMI (1.6%)

Number of cases in each category of wholegrain product consumption at the time of diagnosis are provided in Supplementary Table 6

*ER+/PR+ * oestrogen receptor-positive/progesterone receptor-positive, *ER-/PR-* oestrogen receptor-negative/progesterone receptor-negative, *NNR* Nordic Nutrition Recommendations

Model 1: age-adjusted only

Model 2: multivariable adjusted model, with adjustments for: age, education level (≤ 9 years, 10–11 years, or ≥ 12 years), smoking status (never, former, and current), walking/cycling (< 20 min/day, ≥ 20 and < 60 min/day, or ≥ 60 min/day), exercise (≤ 1 h/week, 2–3 h/week, or ≥ 4 h/week), body mass index (BMI) (< 25 kg/m^2^, ≥ 25 and < 27.5 kg/m^2^, ≥ 27.5 and < 30 kg/m^2^, or ≥ 30 kg/m^2^), family history of breast cancer (yes/no), alcohol consumption (continuous, g/day), consumption of refined grain foods (continuous, g/day), modified Mediterranean Diet Score (mMDS) (low (0–2 points), medium (3–4 points), or high (5–6 points)), and energy intake (continuous, kcal/day)

Model 3: same as Model 2 with additional adjustments for: age at menarche (≤ 12 years, ≥ 13 years), parity (nulliparous, 1–2 children, ≥ 3 children), age at first birth (nulliparous, < 31 years, ≥ 31 years), ever use of hormone replacement therapy (yes/no), ever use of contraceptive pills (yes/no), menopause status and age of occurrence (pre-menopausal, post-menopausal and occurred at < 51 years, post-menopausal and occurred at ≥ 51 years)

^a^Consumption of wholegrain products was determined based on reported consumption of “ready-to-eat” wholegrain food products

## Discussion

In this population-based cohort of primarily postmenopausal women, long-term wholegrain intake in line with the NNR2023 guidelines of ≥ 90 g/day (i.e., full adherence) was associated with lower risk of total breast cancer. An indication of an inverse association was further observed for ER+/PR+ breast cancer, but not ER-/PR-. We observed that different wholegrain products may be differently associated with breast cancer risk: moderate consumption of breakfast cereal (> 0 to < 2 servings/week) was associated with lower risk of total breast cancer risk, whilst high crispbread consumption (≥ 14 servings/week) was associated with higher risk of ER-/PR- breast cancer only.

Previous studies have reported inconsistent results. An inverse association between intake of > 38 g/day and total breast cancer was observed in the Framingham Offspring cohort [9], which, in terms of relative wholegrain intake within a specific population, is in line with the present study, although not when considering absolute values, given 38 g/day is below the median in the present population, but highest in the US study. In contrast, a study in the Danish Diet, Cancer and Health cohort found no association between the highest quartile of intake (39–100 g/day of wholegrain as an ingredient) and total breast cancer risk, or ER+ and ER- subtypes [6], although this study considered wholegrain wheat and rye only. Other studies have investigated consumption of wholegrain foods. Among postmenopausal Danish women, high consumption (median 112 g/day) was not associated with total breast cancer or subtypes [7]. Similarly, in the Nurses’ Health Study II cohort, there was no association among postmenopausal women, however, a lower risk of premenopausal breast cancer was observed among women with the highest wholegrain food consumption (median 1.5 servings/day) [10]. In contrast, an indication of higher risk of total breast cancer was observed in the Iowa Women’s Health cohort among high consumers (19–109 servings/week) [27]. Finally, in a meta-analysis, high consumption of wholegrain food was inversely associated with risk of total breast cancer in case–control but not in cohort studies [11], although these findings may be limited due to challenges in harmonising wholegrain intake/wholegrain product consumption across the included studies, which includes different populations with different wholegrain product composition and varying amounts of wholegrain consumed.

With respect to specific wholegrain products, in a Danish study no association between any wholegrain product and total breast cancer risk was observed [7], with further comparison of the direction/magnitude of point estimates not possible since specific results were not shown. Two US studies also observed no association between any wholegrain product (including e.g., oatmeal, breakfast cereals, and sweetened rye bread) and breast cancer risk [9, 10]. However, in line with the present study, high rye bread consumption (including crispbread) was associated with higher risk of total breast cancer among postmenopausal Icelandic women, whilst high oatmeal consumption was associated with a lower risk [28].

There are multiple potential mechanisms through which wholegrains may be related to breast cancer risk, including anti-inflammatory and immunomodulatory properties, as well as other anti-carcinogenic properties [29, 30]. Wholegrains are a concentrated source of dietary fibre, high intake of which has been previously demonstrated to result in lower levels of pro-inflammatory mediators including interleukin-6 and C-reactive protein [31, 32]. In addition, due to the high fibre content, wholegrain consumption can improve the composition of the gut microbiota [33], thereby increasing the production of short-chain fatty acids and subsequent regulation of the immune response, for instance by preventing synthesis and release of inflammatory mediators such as tumour necrosis factor [34], which has an important role in cancer development [35]. In addition, due to the fibre content, wholegrains may reduce levels of circulating steroid hormone levels [36, 37], particularly oestrogen, which under some circumstances exhibits proliferative effects [38]. However, the function of different wholegrain components is likely impacted by both individual-level microbiota [39, 40] and hormone status [41]. For instance, lignans compete with endogenous oestrogen to inhibit its activity [41] but can also elicit antagonistic effects, depending on the concentration of both lignan and endogenous oestrogen [42].

The differences in direction of point estimates observed between different wholegrain products may be partially explained by the different amount, types, and availability of fibre in different grains, as well as the concentration and bioavailability of different bioactive compounds [12]. For instance, when compared to rye and wheat, oats contain less total dietary fibre and have a higher proportion of soluble fibres compared to insoluble fibres [12]. However, compared to both rye and wheat, oats are high in β-glucan [12] which exhibits antiproliferative and antimutagenic effects [43], which may in part explain the indication of an inverse relationship primarily observed between oat-based products and total breast cancer. Alternatively, by-products of industrial food processing may mask the beneficial actions of some wholegrain properties. For example, crispbread is a major source of acrylamide—classified as a probable human carcinogen by the International Agency for Research on Cancer (IARC) [44]—in a typical Swedish diet [45], with an indication of an association between acrylamide and ER-/PR- breast cancer previously reported [46, 47].

### Strengths and limitations

The strengths of this study include the time-updated analysis, a large sample size, detailed information on covariates at both time points, the use of registry data with essentially complete case ascertainment, and a large number of breast cancer cases. The use of a validated FFQ (with good validity determined [18]) ensures reliability of dietary assessment, although as with all self-reported assessments, the potential of misreport due to recall or social desirability bias remains. As such, potential misclassification of participant dietary data (and hence the exposure) is an important limitation to consider. Further limitations include the assumption of missing dietary data as non-consumption. In addition, whilst we are confident that the most important measures have been adjusted for, we cannot exclude the possibility of residual confounding from unmeasured variables (e.g., genetic factors).

### Future perspectives

There are several practical challenges worth consideration when studying wholegrain intake. These include the different sources of wholegrain under study (e.g., inclusion/exclusion of wholegrain intake from low-wholegrain foods such as some biscuits or pies for which wholegrain options are not usually considered), the exposure to be assessed (e.g., wholegrain intake based on grams of wholegrain or consumption of wholegrain products, and if the latter, how to classify a wholegrain product [20]); the unit of choice (e.g., servings or grams); and what constitutes “high” or “low” intake, which varies between populations. Additionally, in the context of public health, recommendations on wholegrain intake can be challenging to interpret, with clarity on whether the recommendation refers to wholegrain as an ingredient or wholegrain products in general often lacking. A wholegrain intake of 90 g/day could be achieved with consumption of—for instance—the following “ready-to-eat” wholegrain products: 200 g oatmeal (36 g wholegrain), 40 g breakfast cereal (14 g wholegrain), 40 g soft wholegrain bread (14 g wholegrain), and 30 g crispbread (26 g wholegrain), however this may also vary depending on the type of wholegrain products available in different countries/contexts. As such, when considering total wholegrain in the diet, we have reported wholegrain intake as opposed to total consumption of wholegrain products, which we believe to be a strength. When we have investigated more granularly, looking at individual consumption of wholegrain products, we have reported in servings/week to facilitate the public health interpretation. Furthermore, we have reported the main grain type for each of the cereal-based foods. Together, these factors ensure both comparability and repeatability of the present study.

## Conclusion

Wholegrain intake in line with NNR2023 guidelines of ≥ 90 g/day may contribute to lower breast cancer risk. However, different wholegrain products may be differently associated with breast cancer risk, particularly for hormone-receptor subtypes. Thus, further work is needed to elucidate the potential mechanism and relationship. Finally, the findings of this study indicate that future epidemiological studies on wholegrain could benefit from careful consideration of amalgamating wholegrain products into a single entity to examine wholegrain intake, as well as more comprehensive reporting of foods included in a generalised wholegrain term.

## Supplementary Information

Below is the link to the electronic supplementary material.

Supplementary file1 (DOCX 50 kb)

## Data Availability

Data described in the manuscript can be made available upon request to the SIMPLER research infrastructure.

## Abbreviations

ER: Oestrogen receptor
FFQ: Food frequency questionnaire
mMDS: Modified Mediterranean diet score
NNR: Nordic Nutrition Recommendations
PR: Progesterone receptor
SMC: Swedish Mammography Cohort
