# Supplementary material for: Long-term wholegrain intake in line with the Nordic Nutrition Recommendations 2023 and risk of breast cancer in a population-based cohort of women
Source: Eur J Nutr. 2026 Jun 19;65(5):171. doi: 10.1007/s00394-026-04013-8 (PMC13282369; doi:10.1007/s00394-026-04013-8)
Supplement: Supplementary file 1 — Supplementary file1 (DOCX 50 kb) [file 394_2026_4013_MOESM1_ESM.pdf]

Version Date: 26 May 2025

**Supplementary Material**

**Long-term wholegrain intake in line with the Nordic Nutrition Recommendations 2023 and risk of breast cancer in a population-based cohort of women**

## Author Information

Stephanie Pitt[1], Niclas Håkansson[1], Joanna Kaluza[1,2], Susanna C. Larsson[1,3], Agneta Åkesson[1], and Alicja Wolk[1]

[1]Institute of Environmental Medicine, Karolinska Institutet, 17 177, Stockholm, Sweden

[2]Department of Human Nutrition, Warsaw University of Life Sciences–SGGW, 02-787 Warsaw, Poland

[3]Medical Epidemiology, Department of Surgical Sciences, Uppsala University, Uppsala, Sweden

**Supplementary Table 1.** Wholegrain food products from the food frequency questionnaires (FFQs) of the Swedish Mammography Cohort included in the present study.

| Wholegrain food product | Main grain type | Wholegrain per 100 g of dry matter (g)[a] | Wholegrain per 100 g of prepared food (g)[b] |
|---|---|---|---|
| **1997 FFQ** | | | |
| Oatmeal | Oat | 95 | 18 |
| Breakfast cereals[c] | Wheat | 45 | 45 |
| Soft wholegrain bread | Wheat and rye | 50 | 34 |
| Crispbread | Rye | 92 | 87 |
| **2009 FFQ** | | | |
| Oatmeal | Oat | 91 | 17 |
| Muesli | Wheat | 86 | 86 |
| Soft wholegrain bread | Wheat and rye | 53 | 36 |
| Crispbread | Rye | 95 | 89 |
| *Also contributing to wholegrain intake in 2009:* | | | |
| Wholegrain rice | Rice | 100 | 38 |
| Wholegrain pasta | Wheat | 54 | 26 |
| Bulgur/cous cous | Wheat | 76 | 37 |

[a]These values were used to classify cereal products as wholegrain food products.

[b]Grams of wholegrain in prepared food refers to the "actual" amount of wholegrain obtainable from a cereal-based food when ready to eat (i.e. prepared).

[c]In 1997, the food item breakfast cereals included traditional cereals (e.g., cornflakes) as well as muesli and granola, which usually contain nuts, seeds, and/or dried fruit. In 2009, this item was split into two food products, with only muesli remaining as a wholegrain food item.

**Supplementary Table 2.** Hazard Ratio (HR) and 95% Confidence Intervals (CI) between non-time-updated wholegrain intake[a] in relation to NNR2023 guidelines and risk of breast cancer by receptor-defined subtype, in a population-based cohort of Swedish women (*n* = 36,479), totalling 602,248 person years, from baseline (01 Jan 1998) to 31 Dec 2016.

| **Wholegrain intake[a] in relation to NNR2023** | | | **Number of cases** | **Model 1** | **Model 2** |
|---|---|---|---|---|---|
| **Level of adherence** | **Percentage of adherence** | **Intake range (g/day)** | | HR (95% CI) | HR (95% CI) |
| | | | *N* = 1,979 | **Total breast cancer** | |
| Low | < 50% | 0 to < 45 | 860 | Ref. | Ref. |
| Partial | ≥ 50% and < 100% | ≥ 45 to < 90 | 939 | 1.00 (0.91, 1.10) | 1.02 (0.92, 1.12) |
| Full | 100% | ≥ 90 | 180 | 0.80 (0.68, 0.93) | 0.80 (0.67, 0.96) |
| | | *p*-trend | | 0.02 | 0.06 |
| | | | *N* = 1,239 | **ER+/PR+** | |
| Low | < 50% | 0 to < 45 | 524 | Ref. | Ref. |
| Partial | ≥ 50% and < 100% | ≥ 45 to < 90 | 600 | 1.06 (0.94, 1.19) | 1.08 (0.95, 1.22) |
| Full | 100% | ≥ 90 | 115 | 0.85 (0.69, 1.03) | 0.86 (0.69, 1.08) |
| | | *p*-trend | | 0.28 | 0.48 |
| | | | *N* = 204 | **ER-/PR-** | |
| Low | < 50% | 0 to < 45 | 83 | Ref. | Ref. |
| Partial | ≥ 50% and < 100% | ≥ 45 to < 90 | 96 | 1.05 (0.78, 1.42) | 1.07 (0.78, 1.47) |
| Full | 100% | ≥ 90 | 25 | 1.13 (0.72, 1.77) | 1.10 (0.66, 1.83) |
| | | *p*-trend | | 0.57 | 0.68 |

[a]Wholegrain intake was determined as the sum of the wholegrain ingredients from several wholegrain food products.
NNR: Nordic Nutrition Recommendations; ER+/PR+: oestrogen receptor-positive/progesterone receptor-positive; ER-/PR-: oestrogen receptor-negative/progesterone receptor-negative.
Person-time (years) per level of adherence: low: 255,037; partial: 279,454; full: 67,757.
Model 1: age-adjusted only.
Model 2: multivariable adjusted model, with adjustments for: age, education level (≤ 9 years, 10-11 years, or ≥ 12 years), smoking status (never, former, and current), walking/cycling (< 20 mins/day, ≥ 20 and < 60 mins/day, or ≥ 60 mins/day), exercise (≤ 1 hr/week, 2-3 hrs/week, or ≥ 4 hrs/week), body mass index (BMI) (< 25 $kg/m^2$, ≥ 25 and < 27.5 $kg/m^2$, ≥ 27.5 and < 30 $kg/m^2$, or ≥ 30 $kg/m^2$), family history of breast cancer (yes/no), alcohol consumption (continuous, g/day), consumption of refined grain foods (continuous, g/day), modified Mediterranean Diet Score (mMDS) (low (0-2 points), medium (3-4 points), or high (5-6 points)), and energy intake (continuous, kcal/day).
Missing data at baseline were included as a missing indicator category for: education (0.3%), smoking status (1.8%), walking/cycling (7.8%), exercise (10.6%), and BMI (3.4%).

**Supplementary Table 3. Complete data analysis.** Hazard Ratio (HR) and 95% Confidence Intervals (CI) between time-updated wholegrain intake in relation to NNR2023 guidelines and risk of total breast cancer in a population-based cohort of Swedish women ($n$ = 22,084), totalling 376,488 person-years.

| **Wholegrain intake[a] in relation to NNR2023** | | | | | **Total breast cancer** ($n$ = 1,220) | |
|---|---|---|---|---|---|---|
| **Level of adherence** | **Percentage of adherence** | **Intake range (g/day)** | **Number of cases** | **Person-time (years)** | **Model 1** HR (95% CI) | **Model 2** HR (95% CI) |
| Low | < 50% | 0 to < 45 | 436 | 132,448 | Ref. | Ref. |
| Partial | ≥ 50% and < 100% | ≥ 45 to < 90 | 665 | 197,453 | 1.01 (0.90, 1.14) | 1.00 (0.88, 1.14) |
| Full | 100% | ≥ 90 | 119 | 46,587 | 0.77 (0.63, 0.95) | 0.74 (0.60, 0.93) |
| | | *p*-trend | | | 0.04 | 0.02 |

[a]Wholegrain intake was determined as the sum of the dry weight of the wholegrain components from several wholegrain food products.
NNR: Nordic Nutrition Recommendations.
Model 1: age-adjusted only.
Model 2: multivariable adjusted model, with adjustments for: age, education level (≤ 9 years, 10-11 years, or ≥ 12 years), smoking status (never, former, and current), walking/cycling (< 20 mins/day, ≥ 20 and < 60 mins/day, or ≥ 60 mins/day), exercise (≤ 1 hr/week, 2-3 hrs/week, or ≥ 4 hrs/week), body mass index (BMI) (< 25 kg/m$^2$, ≥ 25 and < 27.5 kg/m$^2$, ≥ 27.5 and < 30 kg/m$^2$, or ≥ 30 kg/m$^2$), family history of breast cancer (yes/no), alcohol consumption (continuous, g/day), consumption of refined grain foods (continuous, g/day), modified Mediterranean Diet Score (mMDS) (low (0-2 points), medium (3-4 points), or high (5-6 points)), and energy intake (continuous, kcal/day).
Complete data analysis was conducted, meaning that any participant from the main analysis missing in consumption of the following at baseline were excluded – oatmeal (13.3%), breakfast cereals (19.5%), soft wholegrain bread (15.2%), and crispbread (8.2%).
Analysis was time updated at the point of re-investigation in 2009, meaning that all participants that remained in the analysis received an updated value of the exposure and covariates from that timepoint (except for education level and family history of breast cancer). Any missing information at re-investigation was carried forward from the previous investigation.
Missing data at baseline were included as a missing indicator category for: education (0.2%), smoking status (1.2%), walking/cycling (5.8%), exercise (7.7%), and BMI (2.4%).
Missing data in 2009 were included as a missing indicator category for: smoking status (0.5%), walking/cycling (2.1%), exercise (3.0%), and BMI (1.1%).

**Supplementary Table 4.** Hazard Ratio (HR) and 95% Confidence Intervals (CI) between non-time-updated consumption of wholegrain products and risk of breast cancer by receptor-defined subtype, in a population-based cohort of Swedish women (*n* = 36,479), totalling 602,248 person years, from baseline (01 Jan 1998) to 31 Dec 2016.

| **Consumption of wholegrain products[a] (servings/week)** | **Person-years** | **Total breast cancer** (cases *n* = 1,979) | | **ER+/PR+** (cases *n* = 1,239) | | **ER-/PR-** (cases *n* = 204) | |
|---|---|---|---|---|---|---|---|
| | | **Model 1** HR (95% CI) | **Model 2** HR (95% CI) | **Model 1** HR (95% CI) | **Model 2** HR (95% CI) | **Model 1** HR (95% CI) | **Model 2** HR (95% CI) |
| **Oatmeal** | | | | | | | |
| 0 | 266,979 | Ref. | Ref. | Ref. | Ref. | Ref. | Ref. |
| > 0 and ≤ 2 | 195,840 | 0.96 (0.87, 1.06) | 0.98 (0.88, 1.09) | 0.96 (0.85, 1.09) | 0.97 (0.85, 1.11) | 0.90 (0.66, 1.23) | 0.92 (0.67, 1.26) |
| > 2 | 139,429 | 0.86 (0.76, 0.97) | 0.90 (0.79, 1.02) | 0.90 (0.77, 1.04) | 0.92 (0.79, 1.07) | 0.70 (0.48, 1.02) | 0.70 (0.48, 1.04) |
| *p*-trend | | 0.02 | 0.09 | 0.18 | 0.32 | 0.08 | 0.09 |
| **Breakfast cereals** | | | | | | | |
| 0 | 198,559 | Ref. | Ref. | Ref. | Ref. | Ref. | Ref. |
| > 0 and ≤ 2 | 168,408 | 0.87 (0.78, 0.98) | 0.86 (0.76, 0.96) | 0.83 (0.72, 0.96) | 0.82 (0.71, 0.95) | 0.70 (0.49, 1.01) | 0.70 (0.48, 1.01) |
| > 2 | 235,281 | 0.93 (0.84, 1.03) | 0.92 (0.83, 1.03) | 0.89 (0.78, 1.02) | 0.89 (0.78, 1.03) | 0.78 (0.56, 1.07) | 0.81 (0.58, 1.14) |
| *p*-trend | | 0.45 | 0.43 | 0.29 | 0.33 | 0.27 | 0.47 |
| **Soft wholegrain bread** | | | | | | | |
| < 7 | 215,865 | Ref. | Ref. | Ref. | Ref. | Ref. | Ref. |
| ≥ 7 and < 14 | 182,371 | 0.98 (0.88, 1.09) | 0.97 (0.87, 1.08) | 0.97 (0.84, 1.11) | 0.95 (0.83, 1.10) | 1.17 (0.83, 1.66) | 1.22 (0.86, 1.73) |
| ≥ 14 | 204,012 | 0.93 (0.84, 1.04) | 0.93 (0.83, 1.04) | 0.89 (0.77, 1.02) | 0.98 (0.76, 1.01) | 1.14 (0.82, 1.59) | 1.20 (0.84, 1.71) |
| *p*-trend | | 0.17 | 0.21 | 0.07 | 0.07 | 0.49 | 0.34 |
| **Crispbread** | | | | | | | |
| < 7 | 160,911 | Ref. | Ref. | Ref. | Ref. | Ref. | Ref. |
| ≥ 7 and < 14 | 159,435 | 1.03 (0.91, 1.16) | 1.04 (0.92, 1.17) | 1.03 (0.88, 1.20) | 1.03 (0.88, 1.20) | 1.19 (0.79, 1.81) | 1.24 (0.81, 1.88) |
| ≥ 14 | 281,902 | 1.05 (0.94, 1.17) | 1.07 (0.95, 1.20) | 1.09 (0.95, 1.25) | 1.09 (0.94, 1.26) | 1.58 (1.10, 2.26) | 1.64 (1.13, 2.39) |
| *p*-trend | | 0.38 | 0.26 | 0.23 | 0.22 | 0.009 | 0.007 |

[a]Consumption of wholegrain products was determined based on reported consumption of "ready-to-eat" wholegrain food products.
ER+/PR+: oestrogen receptor-positive/progesterone receptor-positive; ER-/PR-: oestrogen receptor-negative/progesterone receptor-negative; NNR: Nordic Nutrition Recommendations.
Model 1: age-adjusted only.
Model 2: multivariable adjusted model, with adjustments for: age, education level (≤ 9 years, 10-11 years, or ≥ 12 years), smoking status (never, former, and current), walking/cycling (< 20 mins/day, ≥ 20 and < 60 mins/day, or ≥ 60 mins/day), exercise (≤ 1 hr/week, 2-3 hrs/week, or ≥ 4 hrs/week), body mass index (BMI) (< 25 $kg/m^2$, ≥ 25 and < 27.5 $kg/m^2$, ≥ 27.5 and < 30 $kg/m^2$, or ≥ 30 $kg/m^2$), family history of breast cancer (yes/no), alcohol consumption (continuous, g/day), consumption of refined grain foods (continuous, g/day), modified Mediterranean Diet Score (mMDS) (low (0-2 points), medium (3-4 points), or high (5-6 points)), and energy intake (continuous, kcal/day). Missing data at baseline were included as a missing indicator category for: education (0.3%), smoking status (1.8%), walking/cycling (7.8%), exercise (10.6%), and BMI (3.4%).

**Supplementary Table 5. Complete data analysis.** Hazard Ratio (HR) and 95% Confidence Intervals (CI) between time-updated consumption of wholegrain products and risk of total breast cancer in a population-based cohort of Swedish women (*n* = 22,084), totalling 376,488 person-years.

| Consumption of wholegrain products[a] (servings/week) | Number of cases | Total breast cancer (*n* = 1,220) | |
|---|---|---|---|
| | | **Model 1** HR (95% CI) | **Model 2** HR (95% CI) |
| **Oatmeal** | | | |
| 0 | 467 | Ref. | Ref. |
| > 0 and ≤ 2 | 469 | 0.92 (0.81, 1.05) | 0.95 (0.83, 1.08) |
| > 2 | 284 | 0.88 (0.75, 1.02) | 0.92 (0.78, 1.07) |
| *p*-trend | | 0.14 | 0.35 |
| **Breakfast cereals** | | | |
| 0 | 218 | Ref. | Ref. |
| > 0 and ≤ 2 | 419 | 0.91 (0.77, 1.07) | 0.91 (0.77, 1.07) |
| > 2 | 583 | 0.99 (0.84, 1.16) | 0.98 (0.84, 1.16) |
| *p*-trend | | 0.46 | 0.49 |
| **Soft wholegrain bread** | | | |
| < 7 | 399 | Ref. | Ref. |
| ≥ 7 and < 14 | 449 | 0.92 (0.81, 1.06) | 0.91 (0.80, 1.05) |
| ≥ 14 | 372 | 0.91 (0.78, 1.05) | 0.89 (0.77, 1.04) |
| *p*-trend | | 0.17 | 0.16 |
| **Crispbread** | | | |
| < 7 | 279 | Ref. | Ref. |
| ≥ 7 and < 14 | 393 | 1.08 (0.92, 1.26) | 1.09 (0.93, 1.27) |
| ≥ 14 | 548 | 1.06 (0.91, 1.22) | 1.06 (0.91, 1.23) |
| *p*-trend | | 0.67 | 0.68 |

[a]Consumption of wholegrain products was determined based on reported consumption of “ready-to-eat” wholegrain food products.
NNR: Nordic Nutrition Recommendations.
Model 1: age-adjusted only.
Model 2: multivariable adjusted model, with adjustments for: age, education level (≤ 9 years, 10-11 years, or ≥ 12 years), smoking status (never, former, and current), walking/cycling (< 20 mins/day, ≥ 20 and < 60 mins/day, or ≥ 60 mins/day), exercise (≤ 1 hr/week, 2-3 hrs/week, or ≥ 4 hrs/week), body mass index (BMI) (< 25 kg/m$^2$, ≥ 25 and < 27.5 kg/m$^2$, ≥ 27.5 and < 30 kg/m$^2$, or ≥ 30 kg/m$^2$), family history of breast cancer (yes/no), alcohol consumption (continuous, g/day), consumption of refined grain foods (continuous, g/day), modified Mediterranean Diet Score (mMDS) (low (0-2 points), medium (3-4 points), or high (5-6 points)), and energy intake (continuous, kcal/day).
Complete data analysis was conducted, meaning that any participant from the main analysis missing in consumption of the following at baseline were excluded – oatmeal (13.3%), breakfast cereals (19.5%), soft wholegrain bread (15.2%), and crispbread (8.2%).
Analysis was time updated at the point of re-investigation in 2009, meaning that all participants that remained in the analysis received an updated value of the exposure and covariates from that timepoint (except for education

level and family history of breast cancer). Any missing information at re-investigation was carried forward from the previous investigation.
Missing data at baseline were included as a missing indicator category for: education (0.2%), smoking status (1.2%), walking/cycling (5.8%), exercise (7.7%), and BMI (2.4%).
Missing data in 2009 were included as a missing indicator category for: smoking status (0.5%), walking/cycling (2.1%), exercise (3.0%), and BMI (1.1%).

**Supplementary Table 6**. Number of breast cancer cases, including receptor-defined subtype, in a population-based cohort of Swedish women (*n* = 36,479) per consumption level across four wholegrain products, at the time of diagnosis.

| Consumption of wholegrain products[a] (servings/week) | | Total breast cancer | ER+/PR+ | ER-/PR- |
|---|---|---|---|---|
| | **Total cases** | **1,979** | **1,239** | **204** |
| **Oatmeal** | | | | |
| 0 | | 827 | 509 | 85 |
| > 0 and ≤ 2 | | 657 | 412 | 68 |
| > 2 | | 495 | 318 | 51 |
| **Breakfast cereals** | | | | |
| 0 | | 646 | 409 | 74 |
| > 0 and ≤ 2 | | 556 | 348 | 55 |
| > 2 | | 777 | 482 | 75 |
| **Soft wholegrain bread** | | | | |
| < 7 | | 778 | 501 | 68 |
| ≥ 7 and < 14 | | 641 | 400 | 74 |
| ≥ 14 | | 560 | 338 | 62 |
| **Crispbread** | | | | |
| < 7 | | 512 | 321 | 41 |
| ≥ 7 and < 14 | | 589 | 365 | 57 |
| ≥ 14 | | 878 | 553 | 106 |

[a]Consumption of wholegrain products was determined based on reported consumption of "ready-to-eat" wholegrain food products.
The cases within each group pertain to those in time-updated analyses.
